# Microsurgical treatment of posterior circulation aneurysms: an institutional experience from Nepal

**DOI:** 10.1097/MS9.0000000000001785

**Published:** 2024-02-05

**Authors:** Mohan Raj Sharma, Susmin Karki, Amit B. Pradhanang, Gopal Sedain

**Affiliations:** Tribhuvan University Teaching Hospital, Institute of Medicine, Kathmandu, Nepal

**Keywords:** low- and-middle-income countries, microsurgical clipping, posterior circulation aneurysms, subarachnoid haemorrhage

## Abstract

**Background:**

Management strategies and outcomes of patients with posterior circulation aneurysms are varied due to uncertainty in the optimal treatment modality and limited experience of neurosurgeons. Data are scarce regarding patients with posterior circulation aneurysms from developing countries. This study aims to describe the clinical characteristics, management strategies and outcome of patients with these aneurysms treated microsurgically in an academic institute in Nepal.

**Methods:**

The clinical records of patients confirmed to have posterior circulation aneurysms treated microsurgically between July 2014 and July 2022 were retrospectively reviewed. Demographic and clinical characteristics, morphometric characteristics of aneurysms, management strategies, postoperative complications, and 1-year outcomes were described.

**Results:**

Out of 220 aneurysms in 190 patients, 20 were posterior circulation aneurysms. The median age of the patients was 43 (15–60) years. All were ruptured aneurysms. The admission Hunt and Hess grades of 18 (90%) patients were less than or equal to III. The posterior inferior cerebellar artery (8) was the commonest location. The postoperative complications rate was 20%, with the overall mortality of 10%. 80% of patients had a favourable outcome at 1-year follow-up.

**Conclusions:**

The patient characteristics and outcome are comparable with those described in the literature from other countries. With an individualized and careful selection strategy, our results are satisfactory despite fewer operations.

## Introduction

Highlights

- All were ruptured aneurysms where; the posterior inferior cerebellar artery being the commonest location.
- Postoperative complications were noted in 20%, while the mortality was 10%.
- The overall number of patients operated for posterior circulation aneurysms is low in our setting.
- With an individualized and careful selection strategy, our results are satisfactory despite fewer operations.

Posterior circulation aneurysms constitute about 5–15% of all intracranial aneurysms^1–3^. These aneurysms have higher rupture and re-rupture rates and poorer outcomes than those in the anterior circulation^4–6^. Surgery for these aneurysms is technically demanding due to a small working space, depth of the surgical field, and proximity to critical vessels, nerves, and the brain stem. The advent of endovascular therapy has revolutionized the practice of cerebrovascular surgery; the most direct effect has been for posterior circulation aneurysms. In resource-limited countries, like Nepal, endovascular treatment is often unavailable or comes at an exorbitant cost to the patients, and hence, microsurgical clipping is still the mainstay treatment^7–9^.

Though many studies are reported on posterior circulation aneurysms from developed countries, very few have been published from South Asia and none from Nepal^10,11^. This study aims to describe the clinical characteristics, management strategies, and outcomes of patients with posterior circulation aneurysms treated with open, microsurgical techniques in a university hospital in Nepal.

## Materials and methods

After approval from the Institutional Review Committee a retrospective review of the prospectively collected data maintained by the Neurosurgery department and hospital records was conducted. We reviewed all patients with posterior circulation aneurysms with microsurgical clipping by a single surgeon (M.R.S.) in our centre from July 2014 to July 2022 was performed. As patients were fewer in number, all the patients in our series were followed up in the outpatient clinic every three to six months with neurological examination and imaging if necessary. Those who did not show up were contacted via telephone and were requested to visit the clinic. Patients with at least 1 year of follow-up were included in the final analysis.

Clinical grading of all patients with confirmed SAH was done using the Hunt and Hess scale at the time of admission. All patients subsequently underwent a computed tomography angiography (CTA) or a four-vessel digital subtraction angiography (DSA), or both to confirm the presence of aneurysm(s) and define their morphometric characteristics. For posterior circulation aneurysms, our policy is to get DSA in all cases for better surgical planning. Patients were routinely admitted to the neurosurgical intensive care unit and managed as per our departmental protocol. The option for endovascular treatment was only available in the country starting in 2019 in a few private hospitals. Thus, prior to 2019, all patients or families with ruptured aneurysms were offered surgery as the treatment option. After 2019, the patients or families were also given the option of endovascular treatment, and those who chose this treatment were excluded from the data analysis. However, the cost of treatment was (still is) beyond reach for many Nepalese even if the aneurysm was better suited for endovascular treatment. Certain patients such as those with significant parenchymal bleed associated with aneurysms were offered immediate surgery. For these reasons, only1 out of 21 patients with posterior circulation aneurysms from 2019 to 2022 underwent endovascular treatment. Our protocol for posterior circulation aneurysms was to perform a delayed surgery (> 10 days after the ictus) to optimize comorbidities and avoid operation on these technically difficult aneurysms in a ‘tight brain’ in the peak period of vasospasm. All but moribund patients were offered surgery. The surgical procedure involved a craniotomy tailored to the aneurysm location, identification, and dissection of the aneurysm, absolute haemostasis, and closure. A follow-up CTA or DSA was obtained in all cases to ensure complete obliteration of the aneurysm either before discharge or at the time of the first follow-up in six weeks. If the angiogram showed complete obliteration, no further angiography was performed.

Apart from demographic and clinical variables, the admission CT, CTA, DSA, and follow-up CTA or DSA were reviewed. Age, sex, Hunt and Hess scale at admission, location and number of aneurysms, the management strategy, immediate postoperative complications, and outcome in one year based on the modified Rankin scale (mRS) were described.

Continuous variables were reported using medians and frequencies, and counts were used to summarize the categorical variables. As our sample size was small, an analysis of risk factors for poor versus good outcomes was not possible. The work has been reported in line with the PROCESS criteria^12^.

## Results

During the study period, 220 cerebral aneurysms were microsurgically clipped in 190 consecutive patients. Out of the 190 patients, 20 patients who underwent microsurgical clipping of posterior circulation aneurysms (10.5%) form the basis of this analysis (Table 1).

**Table 1:** Demographic and clinical characteristics and outcomes of patients with posterior circulation aneurysms

Serial no	Age in years	Sex	Aneurysm location	H-H grade before surgery	Postoperative complications	Follow-up period (months)	Outcome in mRS
1	15	Female	Right P2 segment of PCA	III	None	96	0
2	32	Male	Left P2 segment of PCA	IV (rebleed)	Malignant post-op oedema	NA	6 (day 3 of surgery)
3^a^	45	Male	Left vertebro-PICA	II	Difficulty in swallowing	80	3
4	40	Male	Right P2 segment of PCA	II	None	64	0
5	48	Female	Left vertebro-PICA	II	None	60	1
6	60^b^	Male	Basilar tip	III	VAP	60	2
7	52	Male	Left vertebro-PICA	II	aspiration	58	2
8	39	Female	Right P 2 segment of PICA	II	None	50	1
9	48	Female	Basilar tip	II	None	48	3
10	45	Female	Basilar tips	II	None	46	1
11	60^c^	Female	Right vertebro-PICA	I	None	44	1
12	25	Male	Right P4 segment of PCA	I	None	44	0
13	45	Female	Right P_4_segment of PICA	II	None	44	0
14	31	Female	Right P_3_ segment of PCA	II	None	40	0
15^d^	55	Female	Basilar tip	IV	Ischaemic changes in the brain stem	NA	6 (day 6 of surgery)
16	48	Female	Basilar tip	III	None	36	1
17	44^e^	Female	Right vertebra-PICA	III	None	36	1
18	38	Female	Right P_4_ segment of PCA	I	None	34	1
19	42	Male	Right vertebro-PICA of PCA	II	None	30	1
20	49^e^	Female	Basilar trunk	III	None	30	0

EVD, external ventricular drainage; MCA, middle cerebral artery; mRS, modified Rankin scale; NA, not applicable; PCA, posterior cerebral artery; PCoM, posterior communicating artery; PICA, posterior inferior cerebellar artery; VAP, ventilator associated pneumonia.

a Patient that required a permanent feeding jejunostomy.

b Patient with multiple aneurysms; another aneurysm on the left PCoM.

c Patient with multiple aneurysms; another aneurysm on the right MCA.

d Patient with atherosclerotic aneurysm wall.

e Patients that required preoperative EVD for hydrocephalus.

### Demographic characteristics and clinical features

Patients’ age in this series ranged from 15 to 60 years, with a median of 43 years. There was a female predilection (M:F = 7:13) for the disease. None of the patients had a family history of aneurysms or other vascular diseases. Regarding comorbidities, only two patients had a known history of hypertension and one patient had diabetes mellitus.

As shown in Table 1, the admission Hunt and Hess grades of 18 (90%) patients were less than or equal to III at presentation.

### Fisher grading and modified fisher grading

Table 2 depicts the Fisher and modified Fisher grading of our patient population. Grade 3 represented the higher number of patients in both Fisher and modified Fisher grades.

**Table 2:** Number of patients based on Fisher and modified Fisher grading (*n* =20)

Grade	Fisher, *n* (%)	Modified Fisher, *n* (%)
0	NA	0
1	0	0 (10)
2	4 (30)	6 (20)
3	9 (50)	9 (50)
4	5(20)	5 (20)

NA, not applicable.

### Aneurysm location and morphometric characteristics

Table 1 shows the distribution of aneurysms in different locations in our series. Eighteen patients had exclusively posterior circulation aneurysms, whereas two patients had associated anterior circulation aneurysms as well, which were deemed unruptured based on the location of the haemorrhage. The posterior inferior cerebellar artery (PICA) was the commonest (8) location, followed by the basilar artery (6) and the posterior cerebral artery (PCA) (6).

### Management strategy

**Figure 1:**
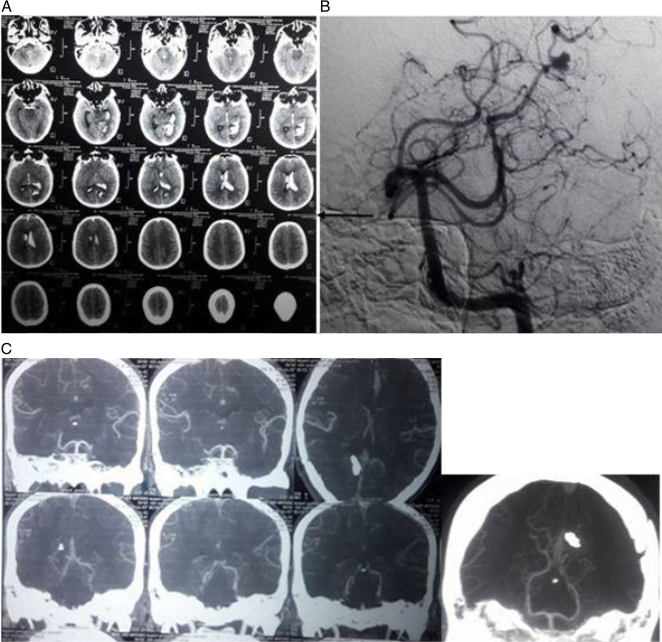
Plain CT head (A) of a 25-year-old male who presented with acute onset severe headache of 24 h duration. Digital subtraction angiography (B) shows a bi-lobed aneurysm in the P4 segment of the left posterior cerebral artery (arrow). This patient underwent trapping of the aneurysm as clip placement without significantly narrowing the parent vessel was impossible during surgery. Postoperative CT angiography (C) shows complete obliteration of the aneurysm. The patient had no neurological complications including intact visual fields postoperatively. CT, computed tomography.

Only one patient underwent surgery on day 5 due to rebleed and acute decrease in the Glasgow Coma Scale. In the remaining patients, the surgery was performed 10 days after the ictus. Microsurgical clipping was performed in 19 patients, and trapping was done in one patient with an aneurysm on the P4 segment of the left PCA (Fig. 1). Two patients underwent preoperative external ventricular drainage (EVD) placement for hydrocephalus.

In this series, all operated patients except those with mortality in the postoperative period underwent postoperative angiography (CTA in 14 and DSA in 4). Complete obliteration of aneurysms was achieved in 16 patients. In two patients, postoperative angiography could not be performed due to early death, and in another two, CTA could not be evaluated due to missing data.

### Postoperative complications and outcome

The follow-up period ranged from 30 to 96 months (mean = 50 months). The postoperative complications included one patient with a permanent feeding jejunostomy due to the loss of gag reflex and nasal regurgitation while swallowing, two patients with third nerve palsy (both following basilar tip aneurysm clipping), and one patient with ventilator-associated pneumonia.

**Figure 2:**
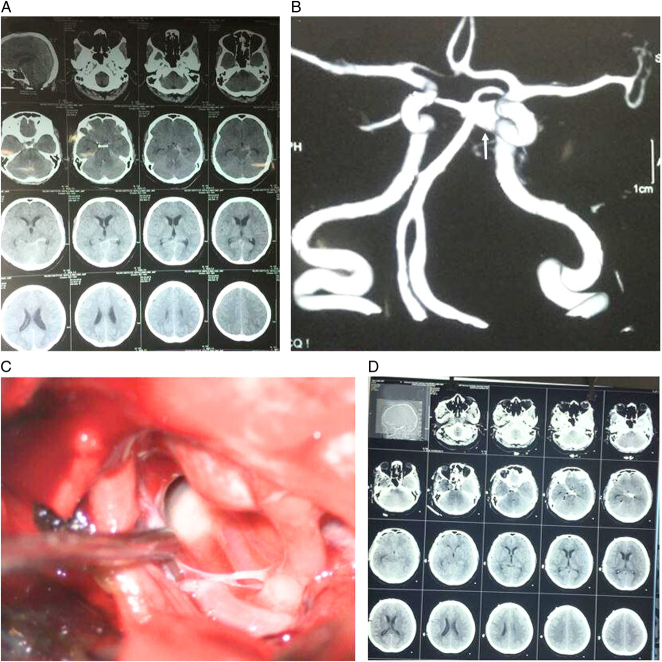
Imaging studies of a 55-year-old woman presenting in the Hunt and Hess grade IV. (A) Plain CT scan shows diffuse subarachnoid haemorrhage. (B) CT angiography reformatted view showing a basilar tip aneurysm with the dome projecting toward the left (arrow). (C) Intraoperative picture showing atherosclerosis of the entire aneurysm, and (D) postoperative CT showing diffuse low density in the brain stem. The patient died 6 days after surgery. CT, computed tomography.

The favourable outcome (mRS 0–2) was achieved in 16 (80%) patients. Two patients had mRS grade 3 in 1 year, and two died in our series. The male patient who died on day 3 of surgery had presented with Hunt Hess grade IV. He had a saccular aneurysm on the P_2_ segment of the left PCA. While in the hospital, he had a second rupture and underwent emergency surgery and subsequently expired. Another patient was a 55-year-old woman with a basilar tip aneurysm who presented on day 2 of the ictus with Hunt and Hess grade IV (Fig. 2). She underwent surgery on day 12. Intraoperatively, we observed that she had severe atherosclerotic disease in the entirety of the aneurysm, including the neck. Despite successful clipping, she never recovered from surgery and died on postoperative day 6. The brain CT obtained 24 h after surgery showed diffuse low-density changes in the brain stem.

As our series is too small, statistical analysis of risk factors for poor outcomes was not possible.

## Discussion

With the publication of the international subarachnoid aneurysm trial (ISAT) results in 2002, there was a paradigm shift in the treatment of cerebral aneurysms^4^. The direct blow was for the posterior circular aneurysms, although the study population largely consisted of good-grade patients with anterior circulation aneurysms. With the publication of the Barrow ruptured aneurysm trial (BRAT) in 2006, there was a resurgence of interest in microsurgery as it included more posterior circulation aneurysms, and results were comparable for anterior circulation aneurysms^6^. BRAT provided evidence of the durability of clips, thus maintaining the viability of open surgery in certain subsets of patients. However, the evidence favored endovascular therapy over microsurgery for posterior circulation aneurysms.

The recommendation may not be entirely generalizable to many low and middle-income countries (LMICs) as the endovascular treatment is either available only in a few urban places or is just too costly^7,8^. Open surgery is the primary or alternative treatment modality of treatment depending on the variety of factors. Whether the patient presents to a modern hospital in an urban setting or to a rural hospital will affect what treatment options are offered. Similarly, the patient’s own financial status will affect what treatment modality is ultimately chosen, as endovascular treatment is considerably more expensive compared to open clipping in LMICs^7,8,11^. The other consideration is aneurysm location, such as the P_1_ segment of PCA or PICA^13^. The publications regarding posterior circulation aneurysms are based on small case series and case reports in the South Asian population^10,14^. Sharma *et al.* ^10^ published the largest series from New Delhi, India on 53 patients from 2002 to June 2011. However, only 19 patients had undergone microsurgery in that series.

Our findings provide some insight into the characteristics and outcomes of patients with posterior circulation aneurysms exclusively treated with microsurgery. In our series, out of all patients operated after aneurysmal SAH, 10.5% had posterior circulation aneurysms, and this is consistent with previously published reports ranging from 5 to 15%^1–3^. In the (ISAT) cohort, only 58 (2.7%) out of 2143 patients had aneurysms in the posterior circulation^4^. In the (BRAT), out of 408 patients, 69 (17%) had posterior circulation aneurysms^6^. In a series of 1000 aneurysms from Pakistan, 3.9% were posterior circulation aneurysms^11^.

### Demographic and clinical characteristics and grades

The demographic characteristics of this study population are similar to those described in the previous series^2,5,15^. The median age is slightly lower than the age reported in the literature. Similar findings were published in the series from India^10^. This probably reflects a shorter life expectancy in the developing world. As in the patients with anterior circulation aneurysms, there is a strong female sex predilection which corroborates with the findings of previous studies^2,15^.

Patients with posterior circulation aneurysms generally present with in poor grades or die before reaching the hospital. However, most patients (18 out of 20) in our series presented with good grades. This could be due to a small number of patients in our study, or the fact that those with poor grades never made it to our institution due to long distance and limited prehospital care. None of the patients in our series had unruptured aneurysms, whereas, in previous publications, ruptured aneurysms constituted between 37 and 81% of cases^5,13,15^.

### Aneurysm location and characteristics

In our series, two patients had multiple aneurysms involving the anterior circulation. The PICA was the commonest location, followed by the basilar tip and middle cerebral artery (MCA). The basilar artery is the most common location of all posterior circulation aneurysms, followed by PCA and PICA^5,13,15^. In the series by You *et al.* ^15^, most aneurysms were located in PCA and PICA. Kim and colleagues, in the South Korean population, reported the basilar artery as the commonest location. Sanai and colleagues, in a large series of 217 patients with 228 posterior circulation aneurysms, reported 106 basilar bifurcation aneurysms^5,13^. In the papers published from India, PICA and basilar artery were the most common locations^10,14^.

### Treatment strategy

Careful, individualized preoperative planning based on angiographic findings is highly desirable. A decision on how to treat these aneurysms microsurgically is based on patient-related (comorbidities, life expectancy, etc.) and aneurysm-related factors as well as the experience and the comfort of the treating surgeon.

In our series, all but one patient underwent delayed surgery. This was deliberate to optimize the patient for surgery (to avoid operating on a tight brain during the peakperiod of vasospasm). The patient who underwent early surgery due to rebleed had an unfavourable outcome.

In our series, 19 aneurysms were clipped, while one P4 segment aneurysm of PCA was trapped. In the series by You *et al.* ^15^, direct clipping was done in 43 (86%) patients, whereas 6 (12%) underwent trapping and bypass.

Complete obliteration of aneurysm was achieved in 16 patients. In the Swiss study, the obliteration rate was 88.5%^2^. In the Indian series by Sharma *et al.* ^10^, the obliteration rate has not been mentioned.

### Postoperative complications and outcome

In general, patients with posterior circulation aneurysms have a less favourable outcome than those with anterior circulation aneurysms^6^. We had 20% postoperative complications that resolved within a year except in one patient who, even after 7 years, had permanent feeding jejunostomy. The commonest complications reported in the published studies are cranial nerve palsies, rebleeding, and neurological worsening due to vasospasm and infarction^5,10,15^.

Two patients in our series required temporary EVD preoperatively. In the study by Maduri *et al.* ^2^, 172 out of 264 (65.1%) required EVD, and 21% ended up with a ventriculo-peritoneal (V-P) shunt. This is unusual as no other studies have reported such a high incidence for cerebrospinal fluid diversion. In the series by You *et al.* ^15^, EVD was put in 2 (4%), and a V-P shunt in 5 (10%) out of 53 patients.

The favourable outcome (mRS 0–2) was achieved in 16 (80%) patients, whereas four (20%) patients had an unfavourable outcome (two deaths and two in grade 3). This result is consistent with regional and global publications on the subject. The mortality of neurosurgical clipping for ruptured intracranial aneurysms was 7.9% in the ISAT cohort^4^. However, as the posterior circular aneurysms represented only 2.7% of total aneurysms, no inference can be drawn regarding the outcome. The BRAT study found a high proportion (63.6%) of unfavourable outcome (mRS >2) in patients undergoing microsurgical clipping for posterior circulation aneurysms in one year^6^. The reported mortality in other series is between 2.7 and 8%^13,15^. In the series by Sharma *et al.* ^10^, 3 patients out of 19 died postoperatively with a mortality of 15.8%. In the series by Maduri and colleagues, a favourable outcome (mRS ≤ 3) was documented in 65.7% (159/264) in one year^2^. However, they included up to mRS grade 3 as favourable outcome.

The 10% mortality in our series warrants clarification. One patient who died in our series experienced rebleed in the hospital and was comatose before surgery. He also had an intraoperative rupture of the aneurysm, requiring prolonged use of a temporary clip. The second patient was in Hunt and Hess grade IV before surgery and had a severely atherosclerotic aneurysm wall. Her postoperative stroke in the brain stem was most likely due to embolization of the atheromas by the clip placement at the neck of the aneurysm.

The strength of our study is that this is the first series of posterior circulation aneurysms reported from Nepal reflecting our patient population and management strategy. Our results, albeit based on a small series, shows that microsurgery is a viable mode of treatment.

Finally, there are a few limitations of our study. This is a retrospective single-centre study of patients operated by a single surgeon, and inherent biases associated with these cannot be ignored. The postoperative obliteration rate was based on CTA in 12 patients in our series, and this may not reflect the true obliteration rate as the diagnostic accuracy of the CTA is slightly inferior to DSA^16^. Given the fewer procedural complications with CTA, our policy was to obtain a CTA. We performed DSA only when the results of the CTA were inconclusive. Also, subgroup analysis and correlation between different factors and outcomes were not possible due to the small number of patients in this study.

## Conclusions

Although a small from a global perspective, this series shows that satisfactory results can be achieved after microsurgery for posterior circulation aneurysms. This has a significant bearing in LMICs where the cost of endovascular treatment is either costly or not available. Further larger multicentric studies in LMICs are encouraged to better define the characteristics and outcome.

## Ethical approval

The approval was given by Institutional Research Committee (IRC) of Institute of Medicine (IOM), Tribhuvan University.

## Consent

As, this is a retrospective case series, which did not require patient involvement directly patient consent was not needed. The patient’s identity has not been disclosed.

## Sources of funding

None.

## Author contribution

M.R.S.: conceptualization, data collection, drafting of the original manuscript, and revision of the manuscript. S.K.: data collection, drafting the original manuscript, revision of the manuscript. S.S., A.P., and G.S.: revision of the manuscript.

## Conflicts of interest disclosure

No competing financial interests.

## Research registration unique identifying number (UIN)

1. Name of the registry: None.
2. Unique Identifying number or registration ID: None.
3. Hyperlink to your specific registration (must be publicly accessible and will be checked): None.

## Guarantor

Dr. Susmin Karki.

## Data availability statement

All the data generated during this study can be accessed through direct communication with the corresponding author and the agreement of all research team members.

## Provenance and peer review

Not commissioned, externally peer-reviewed.
